# Photoacclimation and entrainment of photosynthesis by fluctuating light varies according to genotype in *Arabidopsis thaliana*

**DOI:** 10.3389/fpls.2023.1116367

**Published:** 2023-03-09

**Authors:** Alexandra J. Burgess, Renata Retkute, Erik H. Murchie

**Affiliations:** ^1^ School of Biosciences, University of Nottingham, Loughborough, United Kingdom; ^2^ Department of Plant Sciences, University of Cambridge, Cambridge, United Kingdom

**Keywords:** photosynthesis, acclimation, induction, fluctuating light, entrainment (light), *Arabidopsis thaliana*

## Abstract

Acclimation of photosynthesis to light intensity (photoacclimation) takes days to achieve and so naturally fluctuating light presents a potential challenge where leaves may be exposed to light conditions that are beyond their window of acclimation. Experiments generally have focused on unchanging light with a relatively fixed combination of photosynthetic attributes to confer higher efficiency in those conditions. Here a controlled LED experiment and mathematical modelling was used to assess the acclimation potential of contrasting Arabidopsis thaliana genotypes following transfer to a controlled fluctuating light environment, designed to present frequencies and amplitudes more relevant to natural conditions. We hypothesize that acclimation of light harvesting, photosynthetic capacity and dark respiration are controlled independently. Two different ecotypes were selected, Wassilewskija-4 (Ws), Landsberg erecta (Ler) and a GPT2 knock out mutant on the Ws background (gpt2-), based on their differing abilities to undergo dynamic acclimation i.e. at the sub-cellular or chloroplastic scale. Results from gas exchange and chlorophyll content indicate that plants can independently regulate different components that could optimize photosynthesis in both high and low light; targeting light harvesting in low light and photosynthetic capacity in high light. Empirical modelling indicates that the pattern of ‘entrainment’ of photosynthetic capacity by past light history is genotype-specific. These data show flexibility of photoacclimation and variation useful for plant improvement.

## 1 Introduction

A potential limitation to plant growth under natural conditions is their ability to acclimate to fluctuating light and the speed at which this acclimation occurs. Within natural environments, light intensities constantly fluctuate as a result of changes in solar angle, seasonal variation, passing clouds or movement of overlapping foliage (Burgess et al., 2019; Wang et al., 2020; Burgess et al., 2021). These fluctuations occur over different timescales, although have been shown to occur as rapidly as sub-second (de Langre, 2008; Burgess et al., 2021; Durand et al., 2021). However, natural shade cast by overhead leaves or cloud cover does not only differ in intensity, but also in spectral quality. This shade often has a reduced red: far red ratio and is deficient in photosynthetically active radiation (PAR: 400-700 nm) due to selective filtering by photosynthetic pigments (Murchie and Horton, 1997; Smith et al., 2017). This creates a complex challenge for the photosynthetic machinery, with the signals and pathways underlying response poorly understood. Plants have evolved a number of mechanisms to cope with fluctuations in the light environment. These enable the efficient capture and use of light at low irradiance, and avoid damage to photosynthetic machinery at high irradiance (Walters, 2005; Demmig-Adams et al., 2012; Ruban, 2017). However, leaf photosynthesis does not respond instantaneously to a sudden change in light, and there is often a delay before steady state is reached. The length of this delay is closely linked to the photosynthetic induction state, which is a physiological condition dependent on recent light history (Sassenrath-Cole and Pearcy, 1994; Stegemann et al., 1999). The induction state is dependent on a number of different processes including photoprotection (Hubbart et al., 2012), the activation state of photosynthetic enzymes (Yamori et al., 2012; Carmo-Silva and Salvucci, 2013; Acevedo-Siaca et al., 2020) and stomatal dynamics (Lawson and Blatt, 2014; Long et al., 2022). However, regardless of induction state, the photosynthetic machinery is able to acclimate (termed photosynthetic acclimation or photoacclimation) to differences in the intensity and spectral composition of light.

Although often referred to as a single process, photosynthetic acclimation involves multiple processes across molecular, cellular and anatomical scales that are often distinct (Athanasiou et al., 2010). Acclimation refers to changes in the composition and organization of photosynthetic apparatus and can be broadly split into two processes: developmental acclimation and dynamic acclimation (Walters, 2005). Despite originating from distinct mechanisms, they may overlap in terms of photosynthesis phenotype. Developmental acclimation describes changes to cell size, number and shape and is set early during development. This can arise as different leaves are exposed to varying light levels; as such they optimize photosynthetic efficiency according to the light environment in which they are exposed. Differences in developmental acclimation state can be seen as changes in the characteristics of the light response curve of photosynthesis. Leaves that developed under a higher light level will have a higher maximum photosynthesis rate (*P_max_*). However, leaves that developed under lower light levels will have a lower light compensation point (LCP). This functions to improve carbon gain at low light intensities, resulting in a shorter, but more sensitive, light-limiting state, thus allowing improved exploitation of low light levels and a swift response to any influx of light due to a passing sun fleck or change in light availability (Yin and Johnson, 2000; Burgess et al., 2021). Differences are observed due to changes in chlorophyll concentration, leaf thickness and molecular alterations such as changes in photosystem I (PSI) and photosystem II (PSII) structure and concentration plus changes in photosynthetic enzyme activities (Murchie and Horton, 1997; Walters, 2005).

The second form of acclimation is dynamic acclimation, which can be characterized as structural and biochemical changes in the photosynthetic machinery of a mature leaf Walters and Horton (1994). It involves reversible responses to light, encompassing changes in the expression of genes and concentration of enzymes which result in alterations in phenotype following an irradiance increase or decrease (Müller et al., 2001; Retkute et al., 2015; Townsend et al., 2018). Responses include changes to PSI and PSII levels or structure; changes in the regulation of electron transport components; changes in enzyme concentrations such as Rubisco and ATPase; changes in granal stacking, changes in stomatal conductance and; the chloroplast avoidance/accumulative response (Walters and Horton, 1994; Anderson et al., 1995; Murchie and Horton, 1997; Walters et al., 1999; Walters, 2005; Li et al., 2009; Dyson et al., 2015; Matthews et al., 2018). As such, full dynamic acclimation may take 7 days (Retkute et al., 2015). Dynamic acclimation can also effect the pigment composition found in a leaf, and adaptations to changes in irradiance can manifest themselves as changes in chlorophyll content and ratios. Chlorophyll *a* is found in the reaction center of both photosystems, and its synthesis is dependent on the synthesis of photosystems, whereas chlorophyll *b* is an accessory pigment, part of the antenna complex and therefore is more readily synthesized when light levels drop in an attempt to harvest maximum light (Anderson, 1980; Anderson, 1986; Murchie and Horton, 1997; Walters et al., 1999). Analysis of chlorophyll content and ratios can provide an alternative to analysis of dark respiration rates, which can be difficult to accurately measure (Walters et al., 2004). Dynamic acclimation can also be seen through changes to the light response curve characteristics, particularly the impact on *P_max_*.

The capacity for plants to undergo developmental or dynamic acclimation is species, or genotype, specific (Murchie and Horton, 1997; Watling et al., 1997; Murchie et al., 2005; Athanasiou et al., 2010). Previously studies on *Arabidopsis thaliana* show accession-, or ecotype-, specific differences in acclimation capacity (Athanasiou et al., 2010). This confers plant fitness under fluctuating light with the popular accession Colombia (Col) exhibiting an inability to undergo dynamic acclimation. Furthermore, Athanasiou et al. (2010) identified the gene At1g61800, encoding a glucose-6-phosphate/phosphate translocator- GPT2, as integral to ability to dynamically activate. GPT2 is thought to mediate dynamic acclimation responses *via* metabolic fluxes. It is responsible for the import of glucose-6-phosphate (G6P) from the cytosol into the chloroplast (Kunz et al., 2010). This has the net effect of increasing starch synthesis, resulting in an increase in chloroplastic phosphate concentration, leading to gene expression changes which allow the cell to sense changes in environmental signals (Walters, 2005; Dyson et al., 2015). Whilst *gpt2* knock out mutants grow normally, and demonstrate developmental acclimation (Niewiadomski et al., 2005), they do not exhibit dynamic acclimation (Dyson et al., 2015), which means they can provide a negative control for demonstrating the fitness benefits of dynamic acclimation in *Arabidopsis*.

Both developmental and dynamic acclimation are important under natural conditions but knowledge of how they interact together is poorly understood. Fluctuating light presents a potential challenge to the acclimation process: a high light acclimated leaf will not perform well under low light and vice versa. This is partly as a result of constraints imposed by the anatomy of the leaf (Oguchi et al., 2003), as well as the correlation between photosynthetic capacity and dark respiration (Givnish, 1988; Niinemets and Tenhunen, 1997; Retkute et al., 2015). Therefore, should a plant exploit high light *via* raising *P_max_* and/or should a plant enhance light capture and reduce respiratory loss under low light? There has been extensive research on how plants acclimate to high- and low- light (Anderson, 1980; Anderson, 1986; Demmig-Adams and Adams, 1992; Murchie and Horton, 1997; Yin and Johnson, 2000; Walters et al., 2004; Scheibe et al., 2005; Walters, 2005; Athanasiou et al., 2010; Kunz et al., 2010; Suorsa et al., 2012; Dyson et al., 2015; Retkute et al., 2015), however, few experiments have focused on the effect of controlled fluctuating light (Chabot et al., 1979; Watling et al., 1997; Yin and Johnson, 2000; Vialet-Chabrand et al., 2017; Matthews et al., 2018). Of those that have been carried out, experiments have predominantly used fluctuating light patterns that alternate between a fixed high and low value, with exceptions e.g. Vialet-Chabrand et al. (2017), thus they do not represent the varying irradiances that plants are subject to in the natural environment.

Recent research has focused more on the short term metabolic efficiencies of photosynthesis during light switches without the ‘background’ shifts in long term changes in composition and this has effects on canopy productivity (Long et al., 2022; Souza et al., 2022). During the shift from low to high light, the ‘induction state’ of photosynthesis determines the rate of response and is governed by enzyme activation state and stomatal aperture. The induction state is in turn determined by the length of time spent in high or low light - the leaf ‘light history’ or entrainment. We currently have limited information on genetic variation of entrainment although a recent study showed variation in deactivation state of Rubisco (Taylor et al., 2022). Furthermore, it is understood that the maximum photosynthetic capacity of a plant is dependent on the number of switches between high and low light intensity and the proportion of time spent under each irradiance (Yin and Johnson, 2000; Retkute et al., 2015). For example, the more time recently spent in high light, the faster the induction response due to the persistence of metabolic and physiological processes that favor photosynthesis such as Rubisco activation state and stomatal conductance. Thus both photoacclimation and entrainment of photosynthesis determine the overall plant response. However there have been few approaches to understand how this can be measured or modelled (Retkute et al., 2015). Until recently, producing accurate fluctuations of light were not possible but lightemitting diode- (LED) based growth chambers enable us to subject plants to a predetermined and controlled pattern of irradiance.

This study addresses two unknown aspects of acclimation. We hypothesized that (1) *Arabidopsis* will engage separate high (photosynthetic capacity) and low (light harvesting and respiration) acclimation responses in a fluctuating light regime and (2) the entrainment of induction state differs between *Arabidopsis* genotypes. We approach this by studying contrasting genotypes of *Arabidopsis* that have varying acclimation responses. After transfer to fluctuating light we measured photosynthetic light response curves and chlorophyll composition. We compared these data to our dynamic model of acclimation (Retkute et al., 2015). We show the existence of separate acclimation responses in fluctuating light that optimize light harvesting and quantum yield at low and capacity under high light while rate of entrainment of induction state by light history varies between accessions.

## 2 Materials and methods

### 2.1 Plant growth

*A. thaliana* ecotypes Landsberg erecta (Ler), Wassilewskija (Ws) and a *gpt2-* mutant (Ws WT background) were selected based on their differing abilities to undergo dynamic acclimation (Athanasiou et al., 2010) The seeds were vernalized at 4°C in a water suspension for 48 hours, prior to transfer into 6.5 cm diameter pots containing Levington M3 compost. One week after germination, seedlings were transplanted into individual pots containing 25 g of Levington M3 compost. Plants were cultivated in a Fytoscope 3000 (Photon System Instruments, PSI, Czech Republic) growth chamber, which uses a combination of red and blue LEDs (1:1 ratio throughout on a photon flux basis) plus far-red LEDs (set to a constant 10 *µmol m* ^-2^ s^-1^ throughout the day). The cabinet was set to a 12 hour photoperiod, with a 20°C day temperature, 16°C night temperature and 50% relative humidity; these conditions remained constant throughout the experiment.

### 2.2 Light treatments

Plants were split into two groups and were subject to two different light treatments: Constant light (CL) and fluctuating light (FL). The CL plants were grown under 266 ± 10 *µmol m* ^-2^ *s* ^-1^ for the duration of the experiment, i.e. up to 37 days. This constant pattern included an initial ramp up and final ramp down stage to represent sunrise and sunset, respectively. The FL group was subject to constant light for 28 days and then transferred into a fluctuating light for the remainder of experiment (i.e. 9 days). 9 days was selected to ensure full dynamic acclimation (Retkute et al., 2015). During growth, plants were kept well-watered. The experiment was repeated four times for Ws and *gpt2-* and three times for Ler.

Due to the short growth span of *Arabidopsis*, response to the FL treatment represents a combination of developmental and dynamic acclimation, where growth for the first 28 days was under a constant light.

**Figure 1:**
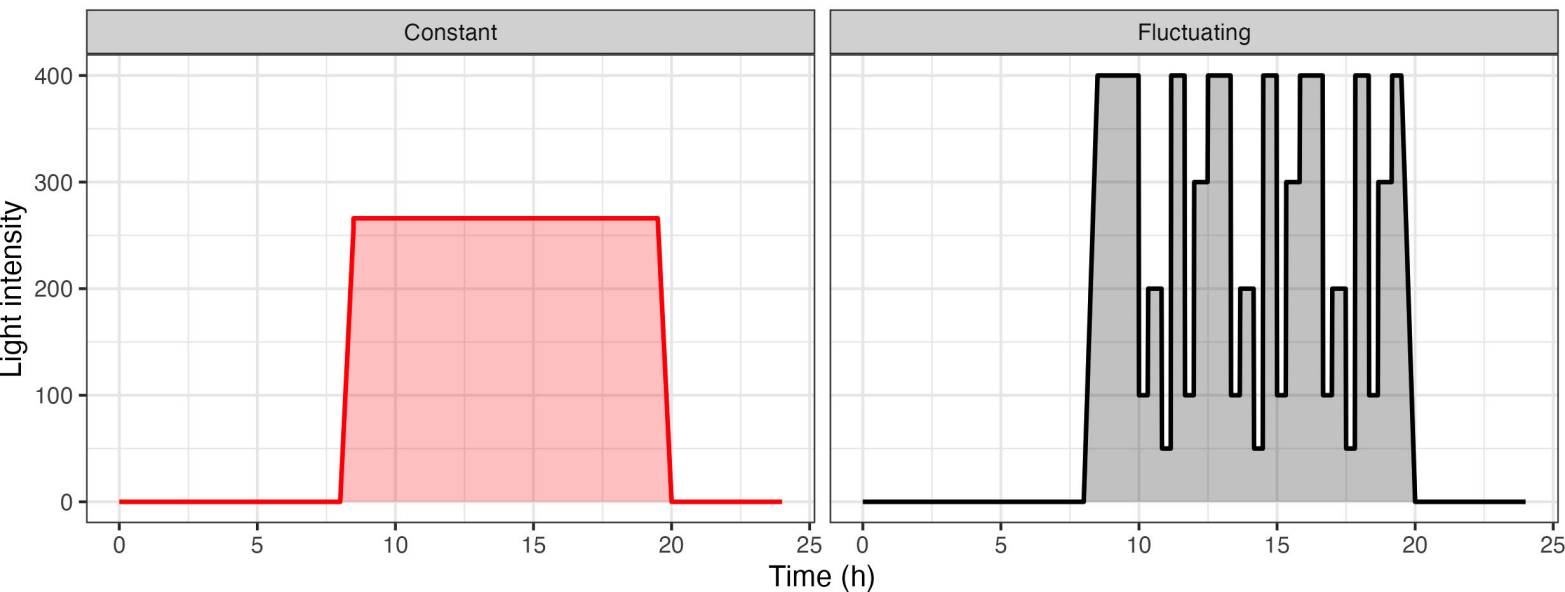
Light patterns used for the analysis of acclimation in *Arabidopsis thaliana*. Plants were split into two groups and were subject to two different light treatments: Constant Light (CL: red) versus Fluctuating Light (FL: black).

The fluctuating light pattern was designed as a re-occurring 3 h 20 min light motif, which was repeated 3 times throughout the day, combined with an initial ramp up and final ramp down phase (**Figure 1**). Each light step was a minimum of 20 min long to discount changes as a result of induction. Care was taken to accommodate different steps in light intensity, decreasing from 400 *µmol m* ^-2^ *s* ^-1^ to 100 *µmol m* ^-2^ *s* ^-1^ or increasing from 50 *µmol m* ^-2^ *s* ^-1^ to 400 *µmol m* ^-2^ *s* ^-1^. Overall, the fluctuating light pattern had the same daily integrated photon dose as the constant light pattern.

### 2.3 Physiological measurements

Both destructive and non-destructive measurements were made on plants. Analysis of rosette area was performed on all plants starting at 21 days after sowing (DAS). Plants were briefly removed from the Fytoscope 3000 growth cabinet into the adjoining room every other working day and were photographed using a RGB camera (Canon EOS 650D SLR, Canon Europa N.V., The Netherlands) using ambient lighting and a scale. Images were analyzed using Image J for rosette area (Schneider et al., 2012).

We assumed that increases in the size of plants followed the exponential growth as a function of time:


(1) $$Area\left(d\right)=a_{0}\exp^{a_{1}d}$$

where *d* is a day from sowing, and *a*_1_ is relative growth rate. Curve fitting for rosette area was carried out using Mathematica (Wolfram, UK).

Following gas exchange measurements (see below), chlorophyll assays were carried out. A size 4 leaf borer was used to take 2 leaf discs per plant from leaves in the 3rd whorl, which were placed immediately in cold, 80% acetone and kept dark. Leaf samples were ground in 80% acetone and made up to 5 ml before being centrifuged for 5 min at 3000 r.p.m, 1600 g. The chlorophyll content and a:b ratios in the supernatant was determined according to (Porra et al., 1989) using absorption with a spectrophotometer at 646.6, 663.6 and 750 *nm*.

### 2.4 Gas exchange measurements

Whole plant light response curves (LRCs) were taken using the LI-COR 6400XT (Li-COR, Nebraska, USA) using the whole plant chamber attachment (6400-17) and RGB Light source (6400-18A for LRCS) at the end of the experiment (36 DAS+). The small size of some of the leaves precluded the use of the standard LI-COR 6400XT chamber, and measurement of the whole plant allows the response of both developmental and dynamic acclimation to be monitored. For all gas exchange measurements, plants were not dark-adapted prior to measurements. The block temperature was maintained at 20°C using a flow rate of 600 l *min^-1^*. For LRCs, light was provided by a combination of in-built red, blue and green LEDs, set to ‘white’ light. Illumination occurred over a series of 12 PAR values between 0 and 1500 *µmol m* ^-2^ *s* ^-1^, with a minimum of two minutes at each light level. At least 6 replicates were taken per experimental repeat for both CL and FL plants.

During the FL treatment period, changes in photosynthesis were measured using the LI-COR 6400XT with the whole plant chamber attachment and sun and sky lid. An ‘autologging’ program was created that took measurements every 15 seconds throughout the FL. The LI-COR was placed inside the Fytoscope chamber, with the chamber providing the light pattern to the individual plant being measured through the sun and sky lid. *CO*_2_ was maintained at 400 p.p.m. throughout. Due to the repeating light signature (3 hours 20 minutes long; see Materials and Methods, Plant Growth), 3 replicates were taken per day on days 1, 3, 5 and 8- post treatment (corresponding to 28, 30, 32 and 35 DAS). Autologging was carried out for two full repeat experiments of the WTs Ler and Ws (i.e. 6 replicates per post treatment day) and one full experiment for the *gpt2-* mutant. The data was normalized according to the average photosynthesis during the last 10 time points at the end of the light pattern and then averaged.

### 2.5 Light response curve fitting

Curve fitting for LRCs was carried out using the Mathematica (Wolfram, UK). The net photosynthetic rate, or assimilation, *A*, as a function of irradiance, *L*, can be described using the non-rectangular hyperbola [41]:


(2) $$A\left(L,P_{max},\alpha ,\theta ,\phi \right)=\frac{\phi L+\left(1+\alpha \right)P_{max}-\sqrt{\left(\phi L+\left(1+\alpha \right)P_{max}{)}^{2}-4\theta \phi L\left(1+\alpha \right)P_{max}\right)}}{2\theta }-\alpha P_{max}$$

The nonrectangular hyperbola is defined by four parameters: the quantum use efficiency (QY), *ϕ*; convexity, *θ*; maximum photosynthetic capacity, *P_max_* and; the rate of dark respiration, *R_d_*. We assumed that the rate of dark respiration is proportional to the maximum photosynthetic capacity, according to the relationship *R_d_* = α*P_max_* (Givnish and Vermeij, 1976; Niinemets and Tenhunen, 1997; Retkute et al., 2015). Fitting was performed using the Mathematica command ‘FindFit’ with a minimum constraint on dark respiration at 0.05 and convexity at 0.8.

### 2.6 Modelling the photosynthetic response

A model incorporating a ‘fading memory’ of the recent light pattern in the form of a time-weighted average for the light was introduced in (Retkute et al., 2015):


(3) $$L_{\tau }\left(t\right)=\frac{1}{\tau }\int_{-\infty }^{t}L\left(t^{\prime }\right)e^{-\frac{t-t^{\prime }}{\tau }}dt^{\prime }$$

This describes the ability for plants to respond more strongly to recent changes in light history. This effectively accounts for photosynthetic induction state, which is very hard to quantify *in situ* as it varies according to the light history of the leaf.

This fading memory was incorporated into the light response functions when calculating instantaneous photosynthetic rate, *P*, at a time *t*. The model was adapted so the time-weighted average was only applied during the transition from low to highlight (to represent induction) but not from high to low light, during which photosynthesis can almost immediately respond.


(4) $$P\left(t,\tau ,P_{max},\alpha ,\theta ,\phi \right)=A\left(\min\;\left(L_{\tau }\left(t\right),L\left(t\right)\right),P_{max},\alpha ,\theta ,\phi \right)$$

We estimated distributions of τ, *P_max_*, α, θ and ϕ for each accession and each day. Parameters were fitted using adaptive multiple importance sampling (Retkute et al., 2021) with likelihood formulated assuming normally distributed errors between photosynthetic rate measured in the experiments and simulated using Eq.4. Parameter prior distributions were assumed to be uniform within following ranges: τ ϵ [0,60], *P_max_* ϵ [0,30], α ϵ [0.03,1], *ϕ* ϵ [0,1] and *θ* ϵ [0.6,0.99].

### 2.7 Statistical analysis

Analysis of variance (ANOVA) was carried out using GenStat for Windows, 17th Edition (VSN International Ltd.). An unbalanced design was used to account for differences in the number of replicates each round (i.e. due to plant mortality). The data was checked to see if it met the assumption of constant variance and normal distribution of residuals. For all statistical analyses, data from each of the lines (Ws, Ler and *gpt2-*) were treated independently because of their differing responses to a change in light. Rosette area was analyzed at 28 DAS and 37 DAS. The former was carried out to ensure that plant growth was same in the CL treatment relative to FL treatment during the period of growth under which they were subject to the same light pattern (grey horizontal line; **Figure 1**) whilst the latter was to determine whether the fluctuating light pattern influenced growth and final rosette area. Chlorophyll *a*:*b* ratio, total chlorophyll content and length of memory fading window were analyzed using ANOVAs.

## 3 Results

### 3.1 Fluctuating light and plant growth

**Figure 2:**
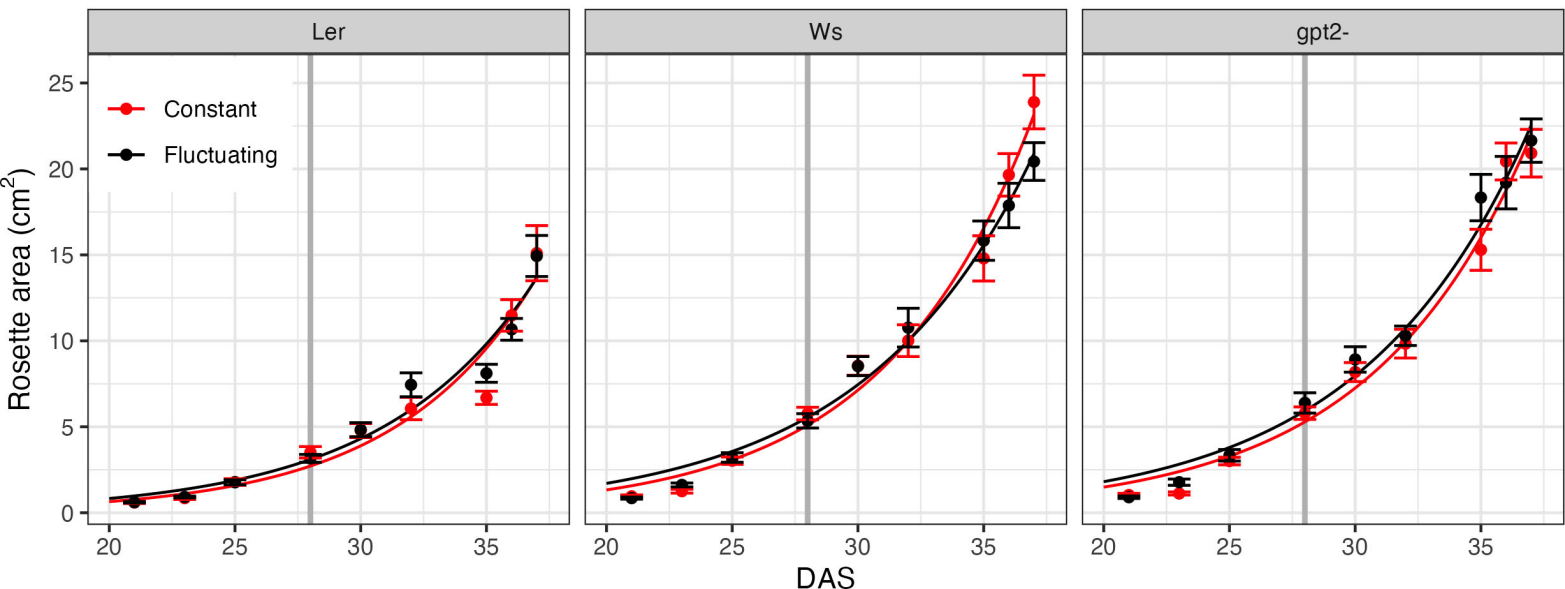
Rosette area time course. Measurements began 21 days after sowing (DAS) for constant light (red) and fluctuating light (black) plants. Each data point correspond to *n*=4 replicates. For the fluctuating light treatment plants, the light pattern was changed at 28 DAS, as denoted by the grey vertical line. Curves show fitted exponential growth given by Eq. 1.

Rosette area of each accession under CL and FL during the course of the experiment is given in **Figure 2**. Whilst this does not account for overlapping leaves or leaf thickness, it can be used as an approximation of growth rate. Two treatment comparisons were performed on each accession in order to see if there were any differences in growth of the plants: first at 28 DAS to ensure consistent growth prior to FL treatment and; secondly at the end of the experiment (37 DAS). At 28 DAS there was no significant difference in rosette area between the treatments in any line, which suggests that up to that point the plants grew similarly. Similarly, at 37 DAS there was no significant difference in the rosette area for any line indicating that 9 days of FL did not significantly alter growth. We estimated the relative [visible] leaf area expansion to be between 5.5 and 6.5 *cm* ^2^ per day (fitted curves in **Figure 2**).

### 3.2 Response of *P_max_* in *Arabidopsis* under fluctuating light

**Figure 3:**
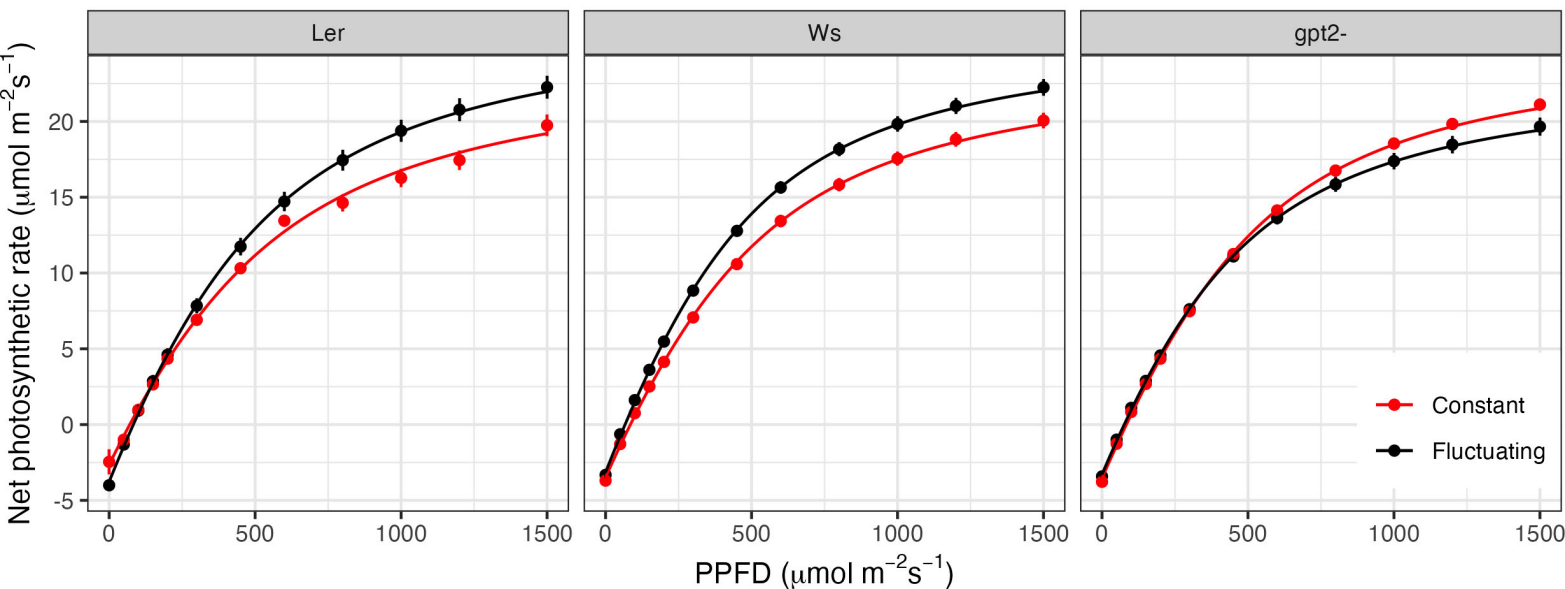
Light response curves for plants grown under constant light (red) versus fluctuating light (black). Light response curves were measured 35 days after sowing, equivalent to 9 days after starting the fluctuating light pattern (FL plants).

LRCs indicate a significant increase in *P_max_* for both the wild type accessions (**Figure 3**: Ws p=0.023; Ler p<0.001), indicative of acclimation to high light. In comparison, the *P_max_* for the *gpt*2- mutant significantly decreased (p=0.013). Direct comparisons between Ws and *gpt*2- under CL showed no significant difference in *P_max_*. However, under FL, *P_max_* in the mutant was significantly lower, indicating the importance of *gpt*2- to FL.

Similar to that seen for *P_max_*, fitted values indicate that for both the WT accessions, QY was significantly higher in plants under FL compared to those under CL (p<0.001 for Ws and p=0.006 for Ler); a further indicator of high light acclimation. There was no significant difference in QY for *gpt*2- under CL versus FL. There was a significant decrease in LCP in the FL plants for Ws (p=0.012) but not Ler or *gpt*2-. A decrease in LCP is an indicator of acclimation to low light. Ler exhibits an increase in *R_d_* in fluctuating light, however, no difference in *R_d_* was found in the other two genotypes.

### 3.3 Change in chlorophyll content and *a*:*b* ratio in *Arabidopsis* under fluctuating light

**Figure 4:**
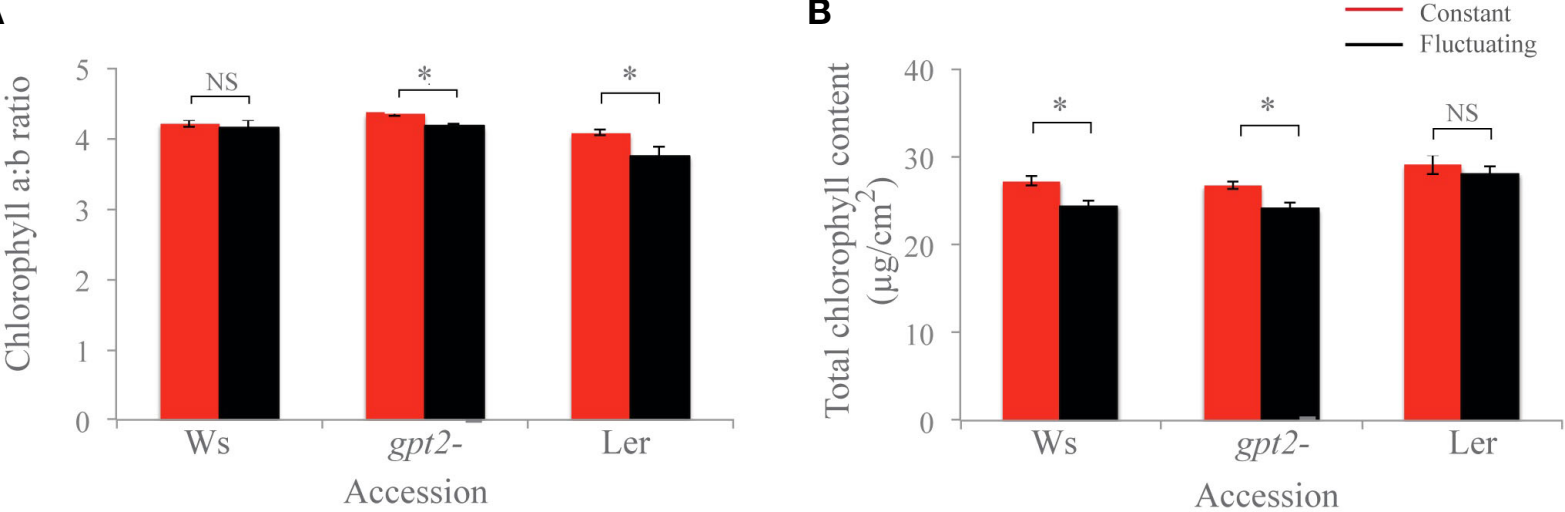
Chlorophyll analysis of plants. **(A)** Chlorophyll a:b ratio. **(B)** Total chlorophyll content. Comparisons were made in each case using an unbalanced ANOVA (NS=Not significant; * = p<0.01). Constant light treatment (red) versus fluctuating light treatment (black).

There was no significant difference in chlorophyll *a:b* in Ws, and for both Ler and *gpt*2- the chlorophyll *a:b* ratios were significantly lower in the FL plants compared to the CL plants (p=0.049 and p=0.004, respectively) (**Figure 4**). Moreover, for both Ws and *gpt*2- the total Chl content was significantly lower in plants under FL compared to those under CL (p<0.001 and p=0.002, respectively). For Ler, no significant change was observed between total Chl amount.

### 3.4 Light history effects on acclimation during fluctuating light

The light motif was split into 8 stages according to the irradiance level (Colored bars; **Figure 4**). For stages 1-4 and 6 (corresponding to the light intensity of 400, 100, 200, 50 and 100 *µmol m* ^-2^ *s* ^-1^, respectively), the average normalized photosynthesis value of the last 50 time points during the step (i.e. at steady state) was calculated. For stage 5, the time taken to reach a normalized photosynthesis value of 0.7, 0.8 and 0.9 was calculated as a proxy for rate of change. There was no significant difference in days post treatment for any of the lines at stages 1-3 and 6. For *gpt*2- and Ws, there was no significant difference during stage 4 (i.e. the step at 50 *µmol m* ^-2^ *s* ^-1^) or 5 (i.e. the step from 50 to 400 *µmol m* ^-2^ *s* ^-1^). However, for Ler there was a significant difference (Stage 4, p= 0.017; Stage 5, p=0.044 and p=0.036 for a normalized photosynthesis of 0.8 and 0.9, respectively). This indicates that in the days following a change in the light environment, Ler was able to respond more quickly to a change in irradiance compared to the other genotypes, thus indicating the importance of the light history.

The time-weighted average (Equation (3) acts as a ‘fading memory’ of the recent light pattern and uses an exponentially decaying weight. If τ = 0 then a plant will able to instantaneously respond to a change in irradiance, whereas if τ< 0 the time-weighted average light pattern will relax over the timescale τ. Previous data from *Arabidopsis* indicates that τ ≈ 0 hours (Retkute et al., 2015). This value of τ (0.3h) represented a maximum leaf ‘memory’ of around 18 minutes that exponentially declines according to time spent in the light.

**Figure 5:**
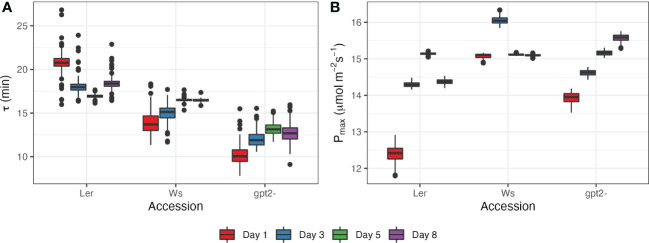
Photosynthetic response at days 1, 3, 5 and 8 after switching to FL regime for the three accessions: **(A)** estimated length of photosynthetic response τ; **(B)** estimated maximum photosynthetic capacity *P_max_*.

For this study, a model, given by Eq.(4), was fit to values of photosynthetic rate during stage 5 of the light motif, i.e. after switching irradiance from 50 *µmol m* ^-2^ *s* ^-1^ to 400 *µmol m* ^-2^ *s* ^-1^ (**Figure 5**; **Supplementary Figure S1**). This time period was selected because it showed the strongest response to change in irradiance levels. There were statistically significant differences between accessions (p<2e^-16^) and between days (p<2e^-16^). Ler had the highest values of the fading memory window, corresponding to the slowest photosynthetic induction (16.9 - 20.8 minutes; **Figure 5A**). Ler plants showed tendency for decrease in τ with more days spend under FL regime, corresponding to an increase in *P_max_* during the latter days of the experiment (**Figure 5B**). The length of photosynthetic response for Ws was estimated to be between 13.8 and 16.5 minutes. The fastest response to increase in light intensity was for *gpt2-*. However, the response time increased with number of days spend under FL for both Ws and *gpt2-* (**Figure 5A**), yet there was still an increase in *P_max_* during the course of the experiment for *gpt2-* but not the WT Ws. Overall, a good correspondence between the fitted model and experimental measurements was found for all accessions and days (**Supplemental Figure S1**).

## 4 Discussion

Photosynthetic acclimation to irradiance is known to include changes in leaf anatomy, biochemistry and physiology (Walters and Horton, 1994; Bailey et al., 2001; Walters, 2005). However, many aspects of the process are still unknown and the regulatory steps underlying acclimation are yet to be fully elucidated (Walters, 2005; Athanasiou et al., 2010; Retkute et al., 2015; Vialet-Chabrand et al., 2017). Transitions from low to high light require photosynthetic induction, including the activation of Rubisco and the opening of stomata (Carmo-Silva and Salvucci, 2013; Lawson and Blatt, 2014), whereas transitions from high to low light require the relaxation of dissipative energy processes, collectively known as non-photochemical quenching (NPQ) (Ruban, 2017; Wang et al., 2020).

### 4.1 Fluctuating light drives independent responses for different acclimation components

**Figure 6:**
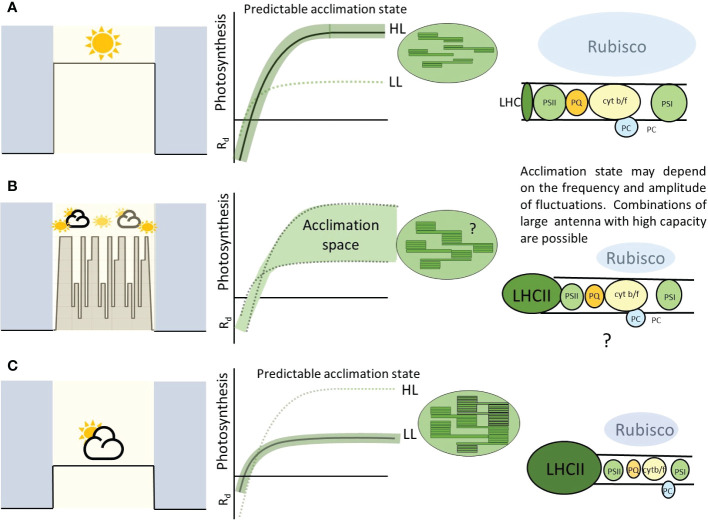
A highly schematic figure to summarize the principles arising from this paper. ‘Square wave’ type growth conditions at a fixed irradiance result in a predictable acclimation condition **(A, C)**. This allows especially high efficiency at these given light levels. Fluctuating conditions, whether natural or imposed, can result in combinations of responses and stoichiometries of the various chloroplast components **(B)**. This is likely to be dependent on the plant, genotype and properties of the imposed light. The cartoon electron transport chain (right pannels) shows the amount of each component proportional with balloon size. In this paper we highlight the possibility of large antenna but high photosynthetic capacity perhaps conferred by higher amounts of electron transport components. This allows the leaf to provide a wider ‘acclimation space’ and exploit high and low light with higher efficiency than **(A)** or **(C)**.

One of the commonly cited functions of acclimation is maintenance of photosynthetic efficiency under the new light regime. For example the lowering of light compensation point, *R_d_* and antenna size under low light. However naturally fluctuations create a dilemma in which both low light and high light acclimation states would be beneficial during the same photoperiod. How do plants deal with this problem? We argue that these features, often seen as fixed to high light or fixed to low light, are not necessarily in conflict (**Figure 6**). Low light acclimated leaves can support high photosynthetic rates as long as photoprotective mechanisms are engaged and other stress factors such as high leaf temperature are not present. Up-regulation of electron transport components can induce a higher *P_max_* during dynamic acclimation without change in Calvin cycle components (Murchie et al., 2005; Vialet-Chabrand et al., 2017).

**Figure 7:**
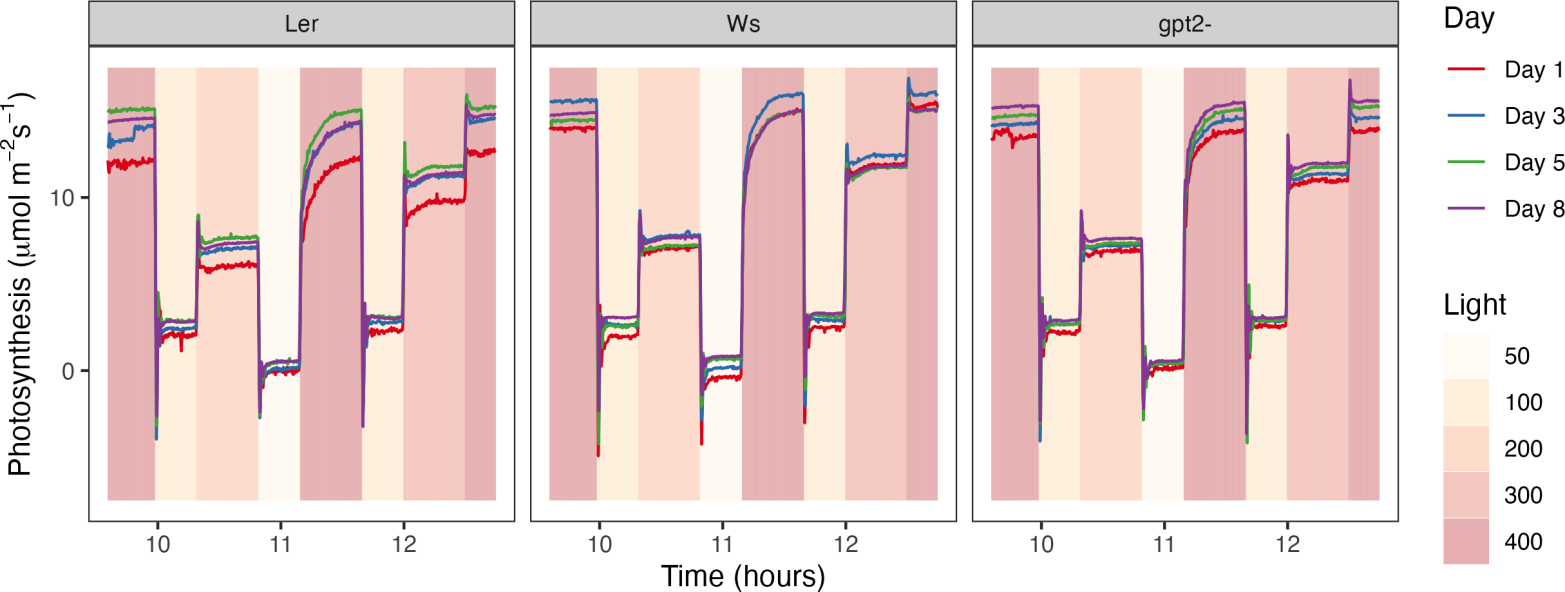
Measured photosynthesis rate in the days following a change in the light pattern. Measurements were made every 15 seconds throughout the light motif at days 1, 3, 5 and 8 post treatment.

In consensus with the literature, both WT plants exhibited an increase in *P_max_* under fluctuation light (**Figure 3**) (Murchie and Horton 1997; Yin and Johnson, 2000; Walters, 2005; Li et al., 2009; Athanasiou et al., 2010; Dyson et al., 2015; Retkute et al., 2015; Vialet-Chabrand et al., 2017). Increasing *P_max_* under high light is usually mirrored by a step-wise increase in Chl *a:b* ratio, due to loss of light harvesting complex (LHCII) (Anderson, 1980; Anderson, 1986; Murchie and Horton, 1997; Bailey et al., 2001; Scheibe et al., 2005). Vialet-Chabrand et al. (2017) found that in the accession Col-0, there was an increase in LHCa1 in non-fluctuating conditions, indicating that fluctuating light was inducing a preferential high light response despite the occurrence of low light periods in the regime. Within this study, a lower Chl *a:b* ratio was observed for Ler in the FL treatment compared to CL treatment (**Figure 7**), this combined with the significant increase in *P_max_* (**Figure 3**) plus a change in photosynthesis at 50 *µmol m* ^-2^ *s* ^-1^ in the days following a change in the light treatment (**Figure 4**) suggests features of acclimation to both the high- and low- light are present (Anderson, 1980; Anderson, 1986; Bailey et al., 2001; Walters et al., 2004) (**Figure 6**). Whilst Chl *a:b* and leaf Chl content were typical of a low light response, *P_max_* was independent of this. With the exception of Ws, the Chl *a:b* responses indicate acclimation of the antenna to low light, not high light upon transfer to FL. Therefore, chlorophyll traits operated independently to the lack of *P_max_* acclimation conferred by the *GPT*2 gene. The reduction in chlorophyll content under FL could therefore have been achieved by a reduction in chloroplast number, size or cell size whilst the stromal fraction for Rubisco content increased to confer the higher *P_max_*. The origin of the higher *P_max_* would therefore need to be determined.

Contrary to previous experiments, there was no significant difference in *R_d_* for any accession (Yin and Johnson, 2000; Dyson et al., 2015), although this may be due to the difficulty in measuring dark respiration within the whole plant chamber. *R_d_* is an essential component of light acclimation and normally rises and falls in line with *P_max_*. A high *R_d_* would be disadvantageous under low light periods.

Bailey et al. (2001) used constant light to identify three distinct phases of acclimation in Ler; a low light response, a high light response and a less pronounced response at intermediate light intensities, which they linked to changes in the content and composition of the thylakoid components as well as both photosystems. This is consistent with the suggestion that the regulation of *P_max_* and Chl *a:b* is largely independent (Bailey et al., 2001). Whilst acclimation to high light was also exhibited by Ws, the corresponding change in chlorophyll was not seen. However, a decrease in Chl *a:b* ratio under FL vs CL was also observed in the *gpt*2- mutants suggesting that the mutant was still able to acclimate to lower light intensities, but not higher intensities. It is unclear why the Ws background would show a different response to *gpt*2- when the *P_max_* shows the typical response to HL. These findings suggest that the effect of fluctuating light on acclimation is to invoke multiple pathways that allow an acclimation response to both high and low irradiances.

Athanasiou et al. (2010) grew plants outdoors in unheated green houses, and found that under naturally fluctuating light, WT Ws had a higher fitness relative to *gpt*2- mutants and WT Col. The same study also showed the inability for Col to dynamically acclimate to an increase in irradiance under controlled conditions (see **Supplementary Figure 1** in Athanasiou et al. (2010)). The fact that Col expresses GP T2 but does not acclimate (Niewiadomski et al., 2005) supports the hypothesis that alternative pathways, not involving GPT2, are necessary for dynamic acclimation under fluctuating light. The role of GPT2 is to translocate G6P into the chloroplast to enable starch synthesis (Kunz et al., 2010). This results in an increased chloroplastic phosphate pool, causing changes in gene expression enabling acclimation to high light. In contrast, *gpt*2- mutants have a higher photosynthesis rate at lower light levels than the parental WT Ws plants (Dyson et al., 2015). However, this improved photosynthetic rate may be a result of developmental acclimation, not dynamic. Nevertheless, combined with the significantly lower Chl *a:b* ratio found within this study, this suggests that acclimation components can operate independently.

So far we have taken a simplistic approach, focusing on antenna size as given by Chl *a:b* and features of the light response curve notably *P_max_* which may be limited by Rubisco, stomatal conductance and electron transport rate depending on conditions. We may consider any component of the chloroplast to be part of acclimation and further work is needed to determine how these operate in relation to each other. For example, changes in Rubisco concentration and activity, along with molecular changes such as cytochrome-b/f activity and LHC and photosystem stoichiometry (Murchie and Horton, 1997; Walters et al., 1999; Yano and Terashima, 2001; Walters et al., 2004; Suorsa et al., 2012). We may determine some general trends, i.e., levels of Cytochrome-b/f, ATPase, plastoquinone and Rubisco will be needed to achieve high *P_max_*, the question is how much. Vialet-Chabrand et al. (2017) showed that election transport was more important than Rubisco in *Arabidopsis* Col-0. There may be much variation in nature: there is evidence that in developmentally acclimated plants, some species do not change their chlorophyll *a:b* ratios (Murchie and Horton, 1997; Zivcak et al., 2014). Furthermore, some changes in chlorophyll *a:b* ratio have been attributed to genes involved in LHCII distribution, which is a known induction n response (Allen and Forsberg, 2001; Depge et al., 2003; Bellafiore et al., 2005; Vainonen et al., 2005; Suorsa et al., 2012; Mekala et al., 2015; Retkute et al., 2015).

### 4.2 ‘Entrainment’ of photosynthetic capacity in fluctuating light is genotype - dependent

In previous work we formulated a mathematical framework of dynamic acclimation that defined the optimal adjustments to net photosynthesis under fluctuating light conditions (Retkute et al., 2015). Applied within this study we describe two key aspects of the acclimation process: first the rate of acclimation itself and second ‘entrainment’ of *P_max_* by the fluctuating light. As described previously, transient high light events induce a ‘fading memory’ which influences both the induction state and the likelihood of inducing acclimation (**Figure 5**). We show a new feature of acclimation in fluctuating light which is the ‘fading memory’ or τ which varies according to genotype. This indicates that there is genetic variation for both speed of response to fluctuating light but also in the sensitivity to which the plants sense and measure light transients.

Previous research on variation among *Arabidopsis* accessions found variation between Ler-0 and Ws-2 in the PSI/PSII ratio, the lateral mobility of the thylakoid membrane and in chlorophyll protein complexes (Kaiser et al., 2020; Wójtowicz and Gieczewska, 2021). In particular, the authors proposed that increased value of the nonphotochemical quenching qN or NPQ reported for Ler-0 under control conditions might limit the capacity for the photosynthetic apparatus to adapt to changing light intensities. NPQ is known to be sensitive to changes in the energy status of the chloroplasts (energy-dependent quenching) and thus presents the most sensitive parameter for the early detection of such changes. Whilst Kaiser et al. (2020) did not assess Ws, they found that certain photosynthetic traits were correlated with the ecological niche to which the accession originated. For example, they found that a reduction in PSII operating efficiency (*ϕ*PSII) under fluctuating light correlates with latitude: with those originating further north exhibiting the lowest *ϕ*PSII. Therefore our conclusion for variation in terms of the timing and rate of onset of acclimation is consistent with the known variation among *Arabidopsis* accessions.

### 4.3 Implication of photosynthetic acclimation for crop plants

Fluctuating light experiments have been performed on plants under natural conditions, these found that the ability to acclimate provided a fitness advantage by optimizing photosynthetic efficiency for a new environment (Athanasiou et al., 2010; Suorsa et al., 2012). However, in these experiments, plants were subject to fluctuations in temperature and humidity as well as light thus entangling photosynthetic acclimation to irradiance from that of temperature or humidity is difficult to achieve. Nevertheless, more realistic representations of the natural environment will be critical for determining the adaptive significance of acclimation and determining the limits placed on plants. However, quantifying the physiological response of plants under environmentally relevant conditions is extremely difficult (Walters, 2005).

Photosynthesis in nature responds largely to fluctuating light in addition to the fixed longer term square waves commonly used for studies in photoacclimation (Poorter et al., 2016; Vialet-Chabrand et al., 2017). Confounding this are species- and genotype-specific differences in plant structure as well as physiological capacity will influence the overall impact of growth conditions on performance (Murchie and Horton, 1997; Athanasiou et al., 2010; Burgess et al., 2017; Burgess et al., 2021). Acclimation is a complex process potentially involving most photosynthetic components in the chloroplast and experimental data indicates that the past light history of a leaf is critical in determining the optimal *P_max_* under a given light level (e.g. **Figure 5**; Retkute et al. (2015)). Whilst this can be controlled or determined relatively easily within small plants with simple structures, such as *Arabidopsis*, knowledge of the past light history is difficult to obtain for larger plants, or crop plants, like rice (*Oryza sativa*) and wheat (*Triticum aestivum*) (Murchie et al., 2002; Murchie et al., 2008; Townsend et al., 2018; Burgess et al., 2021). The complex canopy structure of these plants combined with environmental factors such as weather conditions and wind, cloud or solar movement mean that a given section of leaf within the same plant will be subject to light changes that vary in frequency and longevity (Burgess et al., 2015; Burgess et al., 2017; Burgess et al., 2021). Knowledge of the underlying mechanisms of this process, what fitness advantages acclimation provides and how it could be manipulated will therefore be critical in targeting crops for improved productivity and yield.

## 5 Concluding remarks

In consensus with the literature, our findings suggest that dynamic acclimation to high- and low light are controlled by at least two distinct mechanisms, and that both are utilized in *A. thaliana*. Whilst *GPT*2 is required for high light acclimation, it does not seem to be necessary for low light acclimation. Furthermore, whilst light history influences the capacity to acclimate to a change in irradiance, the length, or speed, of response to light history is also genotype specific. This lays the necessary groundwork for understanding the features of fluctuating light and the speed and direction of multi-faceted responses that provide optimal acclimation state to both high and low light within short time periods.

## Data availability statement

The raw data supporting the conclusions of this article will be made available by the authors, without undue reservation.

## Author contributions

All authors conceived the project. AB performed the physical experimentation whilst RR performed the modelling analysis. RR and AB wrote the article with input from EM. All authors contributed to the article and approved the submitted version.

## References

[B1] Acevedo-Siaca L. G. Coe R. Wang Y. Kromdijk J. Quick W. P. Long S. P. (2020). Variation in photosynthetic induction between rice accessions and its potential for improving productivity. New Phytol. 227, 1097–1108. doi: 10.1111/nph.16454 32124982 PMC7383871

[B2] Allen J. F. Forsberg J. (2001). Molecular recognition in thylakoid structure and function. Trends Plant Sci. 6, 317–326. doi: 10.1016/s1360-1385(01)02010-6 11435171

[B3] Anderson J. M. (1980). Chlorophyll-protein complexes of higher plant thylakoids: distribution, stoichiometry and organization in the photosynthetic unit. FEBS Lett. 117, 327–331. doi: 10.1016/0014-5793(80)80973-2

[B4] Anderson J. M. (1986). Photoregulation of the composition, function, and structure of thylakoid membranes. Annu. Rev. Plant Physiol. 37, 93–136. doi: 10.1146/annurev.pp.37.060186.000521

[B5] Anderson J. M. Chow W. S. Park Y.-I. (1995). The grand design of photosynthesis: Acclimation of the photosynthetic apparatus to environmental cues. Photosynthesis Res. 46, 129–139. doi: 10.1007/bf00020423 24301575

[B6] Athanasiou K. Dyson B. C. Webster R. E. Johnson G. N. (2010). Dynamic acclimation of photosynthesis increases plant fitness in changing environments. Plant Physiol. 152, 366–373. doi: 10.1104/pp.109.149351 19939944 PMC2799370

[B7] Bailey S. Walters R. G. Jansson S. Horton P. (2001). Acclimation of arabidopsis thaliana to the light environment: The existence of separate low light and high light responses. Planta 213, 794–801. doi: 10.1007/s004250100556 11678285

[B8] Bellafiore S. Barneche F. Peltier G. Rochaix J.-D. (2005). State transitions and light adaptation require chloroplast thylakoid protein kinase STN7. Nature 433, 892–895. doi: 10.1038/nature03286 15729347

[B9] Burgess A. J. Durand M. Gibbs J. A. Retkute R. Robson T. M. Murchie E. H. (2021). The effect of canopy architecture on the patterning of ‘windflecks’ within a wheat canopy. Plant Cell Environ. 44, 3524–3537. doi: 10.1111/pce.14168 34418115

[B10] Burgess A. J. Gibbs J. A. Murchie E. H. (2019). A canopy conundrum: Can wind-induced movement help to increase crop productivity by relieving photosynthetic limitations? J. Exp. Bot. 70, 2371–2380. doi: 10.1093/jxb/ery424 30481324

[B11] Burgess A. J. Retkute R. Pound M. P. Foulkes J. Preston S. P. Jensen O. E.. (2015). High-resolution three-dimensional structural data quantify the impact of photoinhibition on long-term carbon gain in wheat canopies in the field. Plant Physiol. 169, 1192–1204. doi: 10.1104/pp.15.00722 26282240 PMC4587458

[B12] Burgess A. J. Retkute R. Pound M. P. Mayes S. Murchie E. H. (2017). Image-based 3d canopy reconstruction to determine potential productivity in complex multi-species crop systems. Ann. Bot., 119(4):517–532. doi: 10.1093/aob/mcw242 28065926 PMC5458713

[B13] Carmo-Silva A. E. Salvucci M. E. (2013). The regulatory properties of rubisco activase differ among species and affect photosynthetic induction during light transitions. Plant Physiol. 161, 1645–1655. doi: 10.1104/pp.112.213348 23417088 PMC3613445

[B14] Chabot B. F. Jurik T. W. Chabot J. F. (1979). Influence of instantaneous and integrated light-flux density on leaf anatomy and photosynthesis. Am. J. Bot. 66, 940–945. doi: 10.1002/j.1537-2197.1979.tb06304.x

[B15] de Langre E. (2008). Effects of wind on plants. Annu. Rev. Fluid Mechanics 40, 141–168. doi: 10.1146/annurev.fluid.40.111406.102135

[B16] Demmig-Adams B. Adams W. W. (1992). Photoprotection and other responses of plants to high light stress. Annu. Rev. Plant Physiol. Plant Mol. Biol. 43, 599–626. doi: 10.1146/annurev.pp.43.060192.003123

[B17] Demmig-Adams B. Cohu C. M. Muller O. Adams W. W. (2012). Modulation of photosynthetic energy conversion efficiency in nature: From seconds to seasons. Photosynthesis Res. 113, 75–88. doi: 10.1007/s11120-012-9761-6 22790560

[B18] Depge N. Bellafiore S. Rochaix J.-D. (2003). Role of chloroplast protein kinase stt7 in LHCII phosphorylation and state transition in chlamydomonas. Science 299, 1572–1575. doi: 10.1126/science.1081397 12624266

[B19] Durand M. Matule B. Burgess A. J. Robson T. M. (2021). Sunfleck properties from time series of fluctuating light. Agric. For. Meteorol 308-309, 108554. doi: 10.1016/j.agrformet.2021.108554

[B20] Dyson B. C. Allwood J. W. Feil R. Xu Y. Miller M. Bowsher C. G.. (2015). Acclimation of metabolism to light in arabidopsis thaliana: the glucose 6-phosphate/phosphate translocator gpt2 directs metabolic acclimation. Plant Cell Environ. 38, 1404–1417. doi: 10.1111/pce.12495 25474495 PMC4949648

[B21] Givnish T. (1988). Adaptation to sun and shade: a whole-plant perspective. Funct. Plant Biol. 15, 63. doi: 10.1071/pp9880063

[B22] Givnish T. J. Vermeij G. J. (1976). Sizes and shapes of liane leaves. Am. Nat. 110, 743–778. doi: 10.1086/283101

[B23] Hubbart S. Ajigboye O. O. Horton P. Murchie E. H. (2012). The photoprotective protein PsbS exerts control over CO2 assimilation rate in fluctuating light in rice. Plant J. 71 (3):402–412. doi: 10.1111/j.1365-313x.2012.04995.x 22413771

[B24] Kaiser E. Walther D. Armbruster U. (2020). Growth under fluctuating fight reveals large trait variation in a panel of arabidopsis accessions. Plants 9, 316. doi: 10.3390/plants9030316 32138234 PMC7154909

[B25] Kunz H. H. Häusler R. E. Fettke J. Herbst K. Niewiadomski P. Gierth M.. (2010). The role of plastidial glucose-6-phosphate/phosphate translocators in vegetative tissues of arabidopsis thaliana mutants impaired in starch biosynthesis. Plant Biol. 12, 115–128. doi: 10.1111/j.1438-8677.2010.00349.x 20712627

[B26] Lawson T. Blatt M. R. (2014). Stomatal size, speed, and responsiveness impact on photosynthesis and water use efficiency. Plant Physiol. 164, 1556–1570. doi: 10.1104/pp.114.237107 24578506 PMC3982722

[B27] Li Z. Wakao S. Fischer B. B. Niyogi K. K. (2009). Sensing and responding to excess light. Annu. Rev. Plant Biol. 60, 239–260. doi: 10.1146/annurev.arplant.58.032806.103844 19575582

[B28] Long S. P. Taylor S. H. Burgess S. J. Carmo-Silva E. Lawson T. Souza A. P. D.. (2022). Into the shadows and back into sunlight: Photosynthesis in fluctuating light. Annu. Rev. Plant Biol. 73, 617–648. doi: 10.1146/annurev-arplant-070221-024745 35595290

[B29] Müller P. Li X.-P. Niyogi K. K. (2001). Non-photochemical quenching. a response to excess light energy. Plant Physiol. 125, 1558–1566. doi: 10.1104/pp.125.4.1558 11299337 PMC1539381

[B30] Matthews J. S. Vialet-Chabrand S. Lawson T. (2018). Acclimation to fluctuating light impacts the rapidity of response and diurnal rhythm of stomatal conductance. Plant Physiol. 176, 1939–1951. doi: 10.1104/pp.17.01809 29371250 PMC5841698

[B31] Mekala N. R. Suorsa M. Rantala M. Aro E.-M. Tikkanen M. (2015). Plants actively avoid state transitions upon changes in light intensity: Role of light-harvesting complex II protein dephosphorylation in high light. Plant Physiol. 168, 721–734. doi: 10.1104/pp.15.00488 25902812 PMC4453798

[B32] Murchie E. H. Horton P. (1997). Acclimation of photosynthesis to irradiance and spectral quality in british plant species: Chlorophyll content, photosynthetic capacity and habitat preference. Plant Cell Environ. 20, 438–448. doi: 10.1046/j.1365-3040.1997.d01-95.x

[B33] Murchie E. H. Hubbart S. Chen Y. Peng S. Horton P. (2002). Acclimation of rice photosynthesis to irradiance under field conditions. Plant Physiol. 130, 1999–2010. doi: 10.1104/pp.011098 12481083 PMC166711

[B34] Murchie E. H. Hubbart S. Peng S. Horton P. (2005). Acclimation of photosynthesis to high irradiance in rice: Gene expression and interactions with leaf development. J. Exp. Bot. 56, 449–460. doi: 10.1093/jxb/eri100 15647315

[B35] Murchie E. H. Pinto M. Horton P. (2008). Agriculture and the new challenges for photosynthesis research. New Phytol. 181, 532–552. doi: 10.1111/j.1469-8137.2008.02705.x 19140947

[B36] Niewiadomski P. Knappe S. Geimer S. Fischer K. Schulz B. Unte U. S.. (2005). The arabidopsis plastidic glucose 6-phosphate/phosphate translocator GPT1 is essential for pollen maturation and embryo sac development. Plant Cell 17, 760–775. doi: 10.1105/tpc.104.029124 15722468 PMC1069697

[B37] Niinemets U. Tenhunen J. (1997). A model separating leaf structural and physiological effects on carbon gain along light gradients for the shade-tolerant species acer saccharum. Plant Cell Environ. 20, 845–866. doi: 10.1046/j.1365-3040.1997.d01-133.x

[B38] Oguchi R. Hikosaka K. Hirose T. (2003). Does the photosynthetic light-acclimation need change in leaf anatomy? Plant Cell Environ. 26, 505–512. doi: 10.1046/j.1365-3040.2003.00981.x

[B39] Poorter H. Fiorani F. Pieruschka R. Wojciechowski T. Putten W. H. Kleyer M.. (2016). Pampered inside, pestered outside? Differences and similarities between plants growing in controlled conditions and in the field. New Phytol. 212, 838–855. doi: 10.1111/nph.14243 27783423

[B40] Porra R. Thompson W. Kriedemann P. (1989). Determination of accurate extinction coefficients and simultaneous equations for assaying chlorophylls a and b extracted with four different solvents: Verification of the concentration of chlorophyll standards by atomic absorption spectroscopy. Biochim. Biophys. Acta (BBA) Bioenergetics 975, 384–394. doi: 10.1016/s0005-2728(89)80347-0

[B41] Retkute R. Smith-Unna S. E. Smith R. W. Burgess A. J. Jensen O. E. Johnson G. N.. (2015). Exploiting heterogeneous environments: Does photosynthetic acclimation optimize carbon gain in fluctuating light? J. Exp. Bot. 66, 2437–2447. doi: 10.1093/jxb/erv055 25788730 PMC4629418

[B42] Retkute R. Touloupou P. Basáñez M.-G. Hollingsworth T. D. Spencer S. E. F. (2021). Integrating geostatistical maps and infectious disease transmission models using adaptive multiple importance sampling. Ann. Appl. Stat 15(4), 1980-1998. doi: 10.1214/21-aoas1486

[B43] Ruban A. V. (2017). Quantifying the efficiency of photoprotection. Philos. Trans. R. Soc. B: Biol. Sci. 372, 20160393. doi: 10.1098/rstb.2016.0393 PMC5566887 28808106

[B44] Sassenrath-Cole G. F. Pearcy R. W. (1994). Regulation of photosynthetic induction state by the magnitude and duration of low light exposure. Plant Physiol. 105, 1115–1123. doi: 10.1104/pp.105.4.1115 12232269 PMC159439

[B45] Scheibe R. Backhausen J. E. Emmerlich V. Holtgrefe S. (2005). Strategies to maintain redox homeostasis during photosynthesis under changing conditions. J. Exp. Bot. 56, 1481–1489. doi: 10.1093/jxb/eri181 15851411

[B46] Schneider C. A. Rasband W. S. Eliceiri K. W. (2012). NIH Image to ImageJ: 25 years of image analysis. Nat. Methods 9, 671–675. doi: 10.1038/nmeth.2089 22930834 PMC5554542

[B47] Smith H. L. McAusland L. Murchie E. H. (2017). Don’t ignore the green light: exploring diverse roles in plant processes. J. Exp. Bot. 68, 2099–2110. doi: 10.1093/jxb/erx098 28575474

[B48] Souza A. P. D. Burgess S. J. Doran L. Hansen J. Manukyan L. Maryn N.. (2022). Soybean photosynthesis and crop yield are improved by accelerating recovery from photoprotection. Science 377, 851–854. doi: 10.1126/science.adc9831 35981033

[B49] Stegemann J. Timm H. C. Küppers M. (1999). Simulation of photosynthetic plasticity in response to highly fluctuating light: An empirical model integrating dynamic photosynthetic induction and capacity. Trees 14, 0145. doi: 10.1007/s004680050219

[B50] Suorsa M. Järvi S. Grieco M. Nurmi M. Pietrzykowska M. Rantala M.. (2012). Proton gradient Regulation5 is essential for proper acclimation of iarabidopsis/i photosystem i to naturally and artificially fluctuating light conditions. Plant Cell 24, 2934–2948. doi: 10.1105/tpc.112.097162 22822205 PMC3426124

[B51] Taylor S. H. Gonzalez-Escobar E. Page R. Parry M. A. J. Long S. P. Carmo-Silva E. (2022). Faster than expected rubisco deactivation in shade reduces cowpea photosynthetic potential in variable light conditions. Nat. Plants 8, 118–124. doi: 10.1038/s41477-021-01068-9 35058608 PMC8863576

[B52] Townsend A. J. Retkute R. Chinnathambi K. Randall J. W. P. Foulkes J. Carmo-Silva E.. (2018). Suboptimal acclimation of photosynthesis to light in wheat canopies. Plant Physiol. 176, 1233–1246. doi: 10.1104/pp.17.01213 29217593 PMC5813572

[B53] Vainonen J. P. Hansson M. Vener A. V. (2005). STN8 protein kinase in arabidopsis thaliana is specific in phosphorylation of photosystem II core proteins. J. Biol. Chem. 280, 33679–33686. doi: 10.1074/jbc.m505729200 16040609

[B54] Vialet-Chabrand S. Matthews J. S. Simkin A. J. Raines C. A. Lawson T. (2017). Importance of fluctuations in light on plant photosynthetic acclimation. Plant Physiol. 173, 2163–2179. doi: 10.1104/pp.16.01767 28184008 PMC5373038

[B55] Walters R. G. (2005). Towards an understanding of photosynthetic acclimation. J. Exp. Bot. 56, 435–447. doi: 10.1093/jxb/eri060 15642715

[B56] Walters R. Horton P. (1994). Acclimation of arabidopsis thaliana to the light environment: Changes in composition of the photosynthetic apparatus. Planta 195, 248–256. doi: 10.1007/bf00199685 8547817

[B57] Walters R. G. Ibrahim D. G. Horton P. Kruger N. J. (2004). A mutant of arabidopsis lacking the triose-phosphate/phosphate translocator reveals metabolic regulation of starch breakdown in the light. Plant Physiol. 135, 891–906. doi: 10.1104/pp.104.040469 15173568 PMC514124

[B58] Walters R. G. Rogers J. J. M. Shephard F. Horton P. (1999). Acclimation of arabidopsis thaliana to the light environment: The role of photoreceptors. Planta 209, 517–527. doi: 10.1007/s004250050756 10550634

[B59] Wang Y. Burgess S. J. Becker E. M. Long S. P. (2020). Photosynthesis in the fleeting shadows: An overlooked opportunity for increasing crop productivity? Plant J. 101, 874–884. doi: 10.1111/tpj.14663 31908116 PMC7064922

[B60] Watling J. R. Ball M. C. Woodrow I. E. (1997). The utilization of lightflecks for growth in four australian rain-forest species. Funct. Ecol. 11, 231–239. doi: 10.1046/j.1365-2435.1997.00073.x

[B61] Wójtowicz J. Gieczewska K. B. (2021). The arabidopsis accessions selection is crucial: Insight from photosynthetic studies. Int. J. Mol. Sci. 22, 9866. doi: 10.3390/ijms22189866 34576029 PMC8465966

[B62] Yamori W. Masumoto C. Fukayama H. Makino A. (2012). Rubisco activase is a key regulator of non-steady-state photosynthesis at any leaf temperature and, to a lesser extent, of steady-state photosynthesis at high temperature. Plant J. 71, 871–880. doi: 10.1111/j.1365-313x.2012.05041.x 22563799

[B63] Yano S. Terashima I. (2001). Separate localization of light signal perception for sun or shade type chloroplast and palisade tissue differentiation in chenopodium album. Plant Cell Physiol. 42, 1303–1310. doi: 10.1093/pcp/pce183 11773522

[B64] Yin Z.-H. Johnson G. N. (2000) Photosynthetic acclimation of higher plants to growth in fluctuating light environments. Photosynthesis Res. 63, 97–107. doi: 10.1023/a:1006303611365 16252168

[B65] Zivcak M. Brestic M. Kalaji H. M. Govindjee (2014). Photosynthetic responses of sun- and shade-grown barley leaves to high light: Is the lower PSII connectivity in shade leaves associated with protection against excess of light? Photosynthesis Res. 119, 339–354. doi: 10.1007/s11120-014-9969-8 PMC3923118 24445618

