# Supplementary figures and images for: Photoacclimation and entrainment of photosynthesis by fluctuating light varies according to genotype in Arabidopsis thaliana
Source: Front Plant Sci. 2023 Mar 9;14:1116367. doi: 10.3389/fpls.2023.1116367 (PMC10034362; doi:10.3389/fpls.2023.1116367)

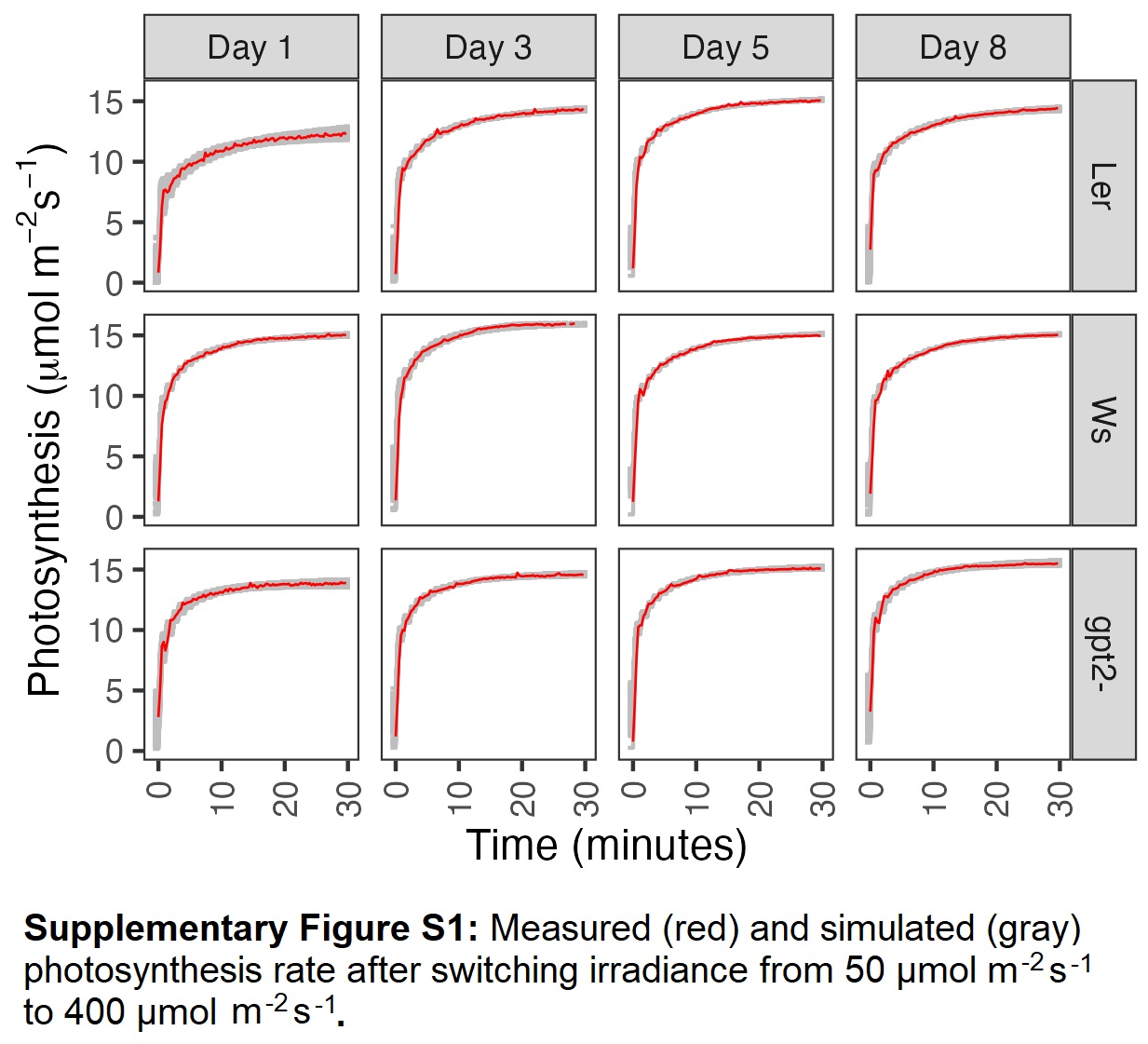

Supplement: Supplementary file 1 [file Image_1.jpg]
